# Crop Nitrogen Retrieval Methods for Simulated Sentinel-2 Data Using In-Field Spectrometer Data

**DOI:** 10.3390/rs13122404

**Published:** 2021-06-19

**Authors:** Gregor Perich, Helge Aasen, Jochem Verrelst, Francesco Argento, Achim Walter, Frank Liebisch

**Affiliations:** 1 Group of Crop Science, Institute of Agricultural Sciences, Department of Environmental Systems Science, ETH Zurich, 8092 Zurich, Switzerland; 2 Image Processing Laboratory (IPL), University of Valencia Science Park, 46980 Valencia, Spain; 3 Water Protection and Substance Flows, Department Agroecology and Environment, Agroscope, 8046 Zürich, Switzerland

**Keywords:** nitrogen, chlorophyll, leaf area index, agro-ecosystem monitoring, spectral indices, random forest, gaussian processes regression, ARTMO toolbox

## Abstract

Nitrogen (N) is one of the key nutrients supplied in agricultural production worldwide. Over-fertilization can have negative influences on the field and the regional level (e.g., agro-ecosystems). Remote sensing of the plant N of field crops presents a valuable tool for the monitoring of N flows in agro-ecosystems. Available data for validation of satellite-based remote sensing of N is scarce. Therefore, in this study, field spectrometer measurements were used to simulate data of the Sentinel-2 (S2) satellites developed for vegetation monitoring by the ESA. The prediction performance of normalized ratio indices (NRIs), random forest regression (RFR) and Gaussian processes regression (GPR) for plant-N-related traits was assessed on a diverse real-world dataset including multiple crops, field sites and years. The plant N traits included the mass-based N measure, N concentration in the biomass (N_conc_), and an area-based N measure approximating the plant N uptake (NUP). Spectral indices such as normalized ratio indices (NRIs) performed well, but the RFR and GPR methods outperformed the NRIs. Key spectral bands for each trait were identified using the RFR variable importance measure and the Gaussian processes regression band analysis tool (GPR-BAT), highlighting the importance of the short-wave infrared (SWIR) region for estimation of plant N_conc_—and to a lesser extent the NUP. The red edge (RE) region was also important. The GPR-BAT showed that five bands were sufficient for plant N trait and leaf area index (LAI) estimation and that a surplus of bands effectively reduced prediction performance. A global sensitivity analysis (GSA) was performed on all traits simultaneously, showing the dominance of the LAI in the mixed remote sensing signal. To delineate the plant-N-related traits from this signal, regional and/or national data collection campaigns producing large crop spectral libraries (CSL) are needed. An improved database will likely enable the mapping of N at the agro-ecosystem level or for use in precision farming by farmers in the future.

## 1 Introduction

### 1.1 Nitrogen in Agro-Ecosystems

Nitrogen (N) plays a pivotal role in the plant life cycle, because it is one of the main nutrients needed for plant biomass production. It is essential for plant metabolism (e.g., chlorophyll) and major plant cell components such as proteins related to crop growth, development and high crop yield performance [1]. N is one of the most abundant molecules in the Earth’s atmosphere [2] and also in plants, where it mainly figures as a building block for the chlorophyll-containing chloroplasts and amino acids that form the plant proteins [3]. Out of all plant proteins, ribulose-1,5-biphosphate carboxylase-oxygenase (rubisco), the major CO_2_-fixing enzyme in plants, is considered to be the most abundant terrestrial protein due to its high concentration in plants [4], which is estimated at about 22% of total leaf N [3]. In a C_3_ plant, 1.7% of total leaf N is allocated to plant chlorophyll and approximately 19% of total leaf N is used in the light harvesting complex [3]. This explains the strong correlation between leaf N and chlorophyll often reported in literature [5]. The concentration of N in plant leaves is, however, relatively small, and ranges from 0.2 to 6.4%, depending on plant species [6].

Plants usually take up N in the form of ammonium (NH_4^+^_) and nitrate (NO_3^−^_) from the soil [7]. This supply is limited and often not sufficient to achieve the desired yield levels in intensive agriculture. Therefore, N is one of the most applied nutrients in the form of fertilizer. This human N input has massively influenced the global N cycle [8], causing negative effects on the agro-ecosystem on regional and global scales [9]. The loss of dissolved nitrate (NO_3^−^_), nitrite (NO_2^−^_) and volatile losses in the form of ammonia (NH_3_) and nitrous oxide (N_2_O) from agricultural systems has been found to pollute ground and surface waters [10,11], deteriorate biodiversity [12], and contribute to greenhouse gas emissions [13,14]. For policymakers, it is therefore becoming increasingly important to know the in- and output of N in agricultural systems at the connection between the field and farm level and the regional watershed, as part of a more holistic approach, at the agroecosystem level [15]. Such information can be used to better manage focus areas such as watersheds with nitrate problems in drinking water reserves. Better-informed spatial recommendations can facilitate targeted application of N input reduction measures at the field and regional scale.

### 1.2 Remote Sensing of Plant Nitrogen and Biomass

#### 1.2.1 On the Terminology of Plant Nitrogen Status

Efficient monitoring of N at both the field and regional level is only possible using remote sensing. In the remote sensing literature, however, many different concepts and terms are used to describe plant ‘N status’ [5]. The used terms include N status, N content, N concentration, plant N, plant N uptake (NUP) or just ‘N’, and they are often used synonymously and are sometimes confused.

The term ‘N status’ is very popular, and often describes the plant N nutrition relative to the optimum desired for the target yield levels in an agronomic scenario [16–18]. This is a result of environmental conditions such as soil available N [19], the plant growth stage [16], and the expected growth performance and yield expectation, and is often used to infer the crops’ fertilizer demand at the field level [20]. It can be evaluated using the plant N concentration (N_conc_), which is the amount of N relative to the dry mass per sampled plant unit (leaf, stems or the whole plant). We therefore consider the N_conc_ to be a massbased N measure.

Opposed to mass-based N measures are area-based N measures, expressed in N per unit area, e.g., kg N ha^−1^ [5]. Such area-based N measures can be obtained by multiplying the N_conc_ with the plant biomass in dry matter [21], whereas the leaf area index (LAI) has also been used to approximate plant biomass on the canopy level [22,23], avoiding growth stage effects in crops’ vegetative growth phase. An area-based N measure represents the total amount of N in the plant, also called nitrogen uptake (NUP), which refers to the total N taken up by the plant. NUP is often measured as ‘aboveground’ NUP, as plant biomass samplings often do not take the root biomass into account [1,20,24,25]. The term NUP is often called ‘N content’ [21]. N content is, however, often used interchangeably with N_conc_, as shown in the review in [5], which can be misleading due to the confusion of mass- and area-based N measures.

The used concepts and terms strongly depend on the perspectives of the ‘end users’ and the anticipated application. For an agronomist, the plant N status is indicative of the plants’ demand for N fertilization applications. The N status can thus either be the N_conc_ or the NUP. In this case, the term ‘N status’ usually refers to NUP as an area-based N measure, since fertilization is usually measured in kg ha^−1^.

For policymakers, the plant N status in agriculture is more likely to focus on the entire agro-ecosystem, especially taking waterways into account [26], i.e., how much N fertilizer has been brought into the agro-ecosystem by farmers? In particular, the risk of N losses, which pose a risk to the environment, and the economic implications thereof, are of interest for environmental stakeholders and policy makers [27]. The concept of N use efficiency (NUE) can be defined as the fraction of N taken up by crops, opposed to the amount available to the plant from soil or fertilizer application [19], and is often used both from an agronomic and from a policymaker’s perspective.

From a remote sensing perspective, the retrieved signal is a proxy for the total N per area (pixel), weighted by its visibility to the sensor. For optical remote sensing, this means that the sunlit top of the canopy has more influence on the retrieved signal than the shaded parts in the lower canopy and consequently, the N in the upper part of the canopy will have higher influence on the retrieved signal than the N in the lower part. Therefore, remotely sensed plant N mostly refers to area-based N information, where the plant biomass is part of the canopy signal.

#### 1.2.2 Remote Sensing of Crop Nitrogen

Remote sensing plant N has often focused on the relationship between the plant leaf chlorophyll content (Chl_AB_) and plant leaf N concentration [18,28,29]. However, the observed relationships have been found to be moderate, with Pearson correlation coefficients being around 0.65 ± 0.15 [30], which can partially be explained by the small amount of leaf N in the light harvesting complex compared to the total leaf N [3,5].

For remote estimation of plant N, spectral information from the red edge (RE) [17,18,24,31,32] and the near infrared (NIR) wavelength [17,24,33] regions have often been used. Studies have often focused on the spectral wavelengths up to 1000 nm [24,33,34]. The short-wave infrared (SWIR) region has not often been used, despite it showing significant potential for plant N estimation [30,35–37]. This spectral region is indicative of nitrogen bonds in amino acids [38], and is thereby more directly connected to N in proteins than the RE spectral region.

Spectral index (SI)-based methods have been in use since the late 1970s [39] and are now widely used in intensive agriculture, e.g., ‘smart farming’ for fertilizer applications, where an increasing number of commercial sensors and remote sensing applications related to plant N exist [32,40–42]. In most studies, a correlation between SIs and the trait of interest is established, and subsequent parametric regression allows prediction of plant N. Today, nonparametric methods such as machine learning regression algorithms (MLRAs), including partial least squares regression (PLSR) [43,44], random forest regression (RFR) [44,45], and Gaussian processes regression (GPR) [46–48], are more frequently used. Methods based on deep learning, such as neural networks, are being explored [49], but are not used frequently [5]. MLRAs and deep learning-based methods are considered nonparametric regression methods [46]. For a more in-depth discussion of commonly used algorithms for N retrieval from remote sensing data, see the review of [5].

#### 1.2.3 Remote Sensing Plant Biomass

The estimation of plant biomass through remote sensing has been extensively performed [30,48,50–54]. Overall, crop traits such as LAI and canopy cover (CC) related to aboveground plant biomass show higher correlations than methods that estimate plant N [48,52]. So far, plant biomass estimation has mostly been based on SIs [17,53,55,56], and MLRAs seem to be less widely in use [48,57]. For biomass traits such as the LAI, mostly information in the RE and NIR regions has been shown to be of importance [52,55,56], with the visible (VIS) region—mainly the red domain—seeing use as well [43,58]. These are similar regions to those shown to be important for the estimation of plant-N-related traits.

#### 1.2.4 Field Spectrometer for Validating Satellite Measurements

Few studies are available for direct plant N estimation using satellite imagery [59,60]. A limitation for such studies is the need for large and expensive field trials for model training and calibration. This is particularly difficult for small-structured agricultural systems such as prevailing in Switzerland. Ref. [61] showed, for average field sizes of 1.6 ha, that no monitoring was possible for up to 22% of the fields because no ‘pure’ field pixel was available at 20-m resolution. Increasing the spatial resolution to 10 m reduced the number of fields that could not be monitored to 6.4%. This issue is exacerbated for Switzerland, where the average farm size is just 21 ha [62] and the field sizes are even smaller (around 1.5 ha, Federal Office of Agriculture census data). Therefore, it would be of great interest if field spectrometer (FS) measurements provided the link between satellite and small-plot N fertilization trials that would otherwise be too small for the calibration of satellite measurements. Thus, several studies have used ground-based FS for the simulation of satellite sensors [24,33,63,64]. In these studies, the FS effectively acted as the tool for a simulation and validation of the data acquired by the satellite sensor.

### 1.3 Aims of This Study

To link satellite imagery with plant traits, data simulation using radiative transfer models (RTMs) has been extensively performed [46,65–67]. Often, if at all, with only small real-world validation datasets. In this paper, we aim to contribute to this research gap by applying parametric and nonparametric methods for estimation of mass- and area-based plant N traits, as well as analysis methods so far only applied to RTM-based studies to a real-world hyperspectral dataset including multiple crops, test sites and years. We further aim to elicit differences between the sensitivity of wavelength regions for plant-N-related traits as a function of bandwidth and number of bands available for prediction in ground-versus satellite-based sensing. We hypothesize that the SWIR region might be of greater importance for N estimation in satellite-based sensing as opposed to ground-based sensing due to the effect of the canopy area in the satellite-based signal. Ultimately, we aimed to estimate the potential of the Sentinel-2 (S2) satellites for plant N estimation in small-scale agricultural agro-ecosystems.

## 2 Materials and Methods

### 2.1 The Dataset

The dataset used in this study originates from three datasets containing spectral libraries (FS reflectance data) of main Swiss field crops (corn, potatoes, sugar beet, summer and winter barley, spring wheat, sunflower and winter wheat) from the years 2013–2016 and 2019. All datasets were collected within the eastern regions of the canton of Zürich, Switzerland. Please see the ‘supplementary materials – dataset’ for more information on the dataset (Figures S1 to S3 and Table S1) as well as a download link for the data used in this study. The 1st and the 2nd datasets originate from FS measurements taken as part of ground truth data collection for the projects SEON (Swiss Earth Observatory Network) [68] and FLOURISH [69]. The 3rd dataset originates from FS measurements on the winter wheat experiments form the work of [20]. In all cases, spectral reflectance data were collected with a FS (ASD FieldSpec4^®^, ASD Inc., Malvern Panalytical, Malvern, UK) with a spectral range of 350–2500 nm resampled to 1 nm band intervals. FS measurements were performed using a white reference and ten measurements distributed in the plot were averaged for each plot. Selected plant traits from this dataset include crop growth stage (BBCH), the mass-based N measure N concentration (N_conc_), chlorophyll_AB_ concentration (Chl_AB_) and LAI as seen in Table 1. To approximate total N and Chl_AB_ on the canopy level, N_conc_ and Chl_AB_ were multiplied with LAI, forming two additional traits: LAI*N_conc_ and LAI*Chl_AB_. The trait LAI*N_conc_ approximates the Nitrogen uptake (NUP), as an area-based N measure. Ref. [22] suggested multiplying LAI with Chl_AB_ to increase canopy level N status estimation.

The mentioned traits were determined on 1 to 4 m^2^ plots located in within farmers’ fields after being evaluated for crop growth stage according to the BBCH scale [70] and FS measurements. Within the same plots, LAI was non-destructively measured with a LI-COR LAI-2200 (2000) Plant Canopy Analyzer (LI-COR Biosciences, Lincoln, NE, USA), as described in detail by [71]. Total biomass samples (very early growth stages) or leaf subsamples of 10 to 20 of the youngest fully developed leaves (later growth stages) were collected in the measurement plots and subsequently dried for N analysis and freeze dried for Chl_AB_. N_conc_ was measured with an elemental analyzer (Flash EA Series, Thermo Fisher Scientific, Waltham, MA, USA) or EURO EA (HEKAtech GmbH, Wegberg, Germany), Chl_AB_ was measured using 95% ethanol extraction and subsequent absorbance measurement by a photometer (EnSpire multimode plate reader, Perkin Elmer, Waltham, MA, USA) at 470, 649 and 664 nm using the equations given in [72].

To test the effect of crop canopy structure on the obtained reflection signal [73], four subsets were created: (1) An ‘erectophile’ dataset containing the crop species with erectophile morphology winter wheat, winter barley, spring wheat and corn, (2) a ‘planophile’ dataset containing the broad-leaved crop species sugar beet, rapeseed, sunflower and potatoes, (3) a dataset containing only winter wheat and (4) a dataset containing only sugar beet (Table 1).

### 2.2 Data Analysis

#### 2.2.1 Dataset Pre-Processing

For the analysis, the atmospheric water absorption bands in the wavelength regions 1350–1440 nm, 1790–1990 nm and 2400–2500 nm were omitted from the FS data. Data in the 350–400 nm region was also omitted due to the low signal-to-noise ratio. To speed up computation, the FS data were resampled into 10-nm intervals (in the following referred to as the FS dataset). All dataset pre-processing and subsequent analysis was performed in R statistical software version 4.0.3 [74]. The R package ‘hsdar’ [75] was used to resample the FS dataset to the spectral resolution of the ‘MultiSpectral Instrument’ (MSI, Table 2) of S2 by using the S2 spectral response function provided by ESA(in the following referred to as S2 dataset).

#### 2.2.2 Normalized Ratio Indices Generation

SIs were calculated as normalized ratio indices (NRIs, Equation (1)) using all possible band combinations of wavelengths *λ_A_* and *λ_B_*:

(1) $$NRI=\frac{\lambda_{A}-\lambda_{B}}{\left(\lambda_{A}+\lambda_{B}\right)}$$

This resulted in 14,706 unique NRI combinations for the FS dataset and 66 combinations for the S2 dataset. The calculated NRIs were correlated against the individual crop traits (Table 1) using Pearson’s correlation. The Pearson’s correlation was squared to obtain the coefficient of determination (R^2^). For the NRI scoring the highest R^2^ value was used to fit a linear regression equation to the trait of interest [24,36,52,76,77].

#### 2.2.3 Random Forest Regression

Random Forest Regression (RFR) was used as a nonparametric machine learning method to regress the individual crop traits on the spectral data. RFR was performed with ten-fold cross validation [44,78]. RFR was implemented using the ‘caret’ [79] and ‘ranger’ [80] packages in R. The optimal model parameter *mtry* (the number of variables to use in each tree) was determined using the best performing model elicited in cross-validation. The RF variable importance scores were calculated using the permutation importance [81] and were used to rank the importance of the available and used spectral bands for the estimation of the trait of interest.

#### 2.2.4 Gaussian Processes Regression–Band Analysis Tool

An alternative spectral analysis was conducted in the automated radiative transfer models operator (ARTMO) toolbox [82]. ARTMO consists of a suite of radiative transfer models and post-processing toolboxes, such as the global sensitivity analysis (GSA) toolbox [83], the MLRA toolbox [84] and the emulator toolbox [85]. The MLRA and emulator toolboxes consist of a suite of MLRAs for mapping applications and subsequent analysis. The Gaussian processes regression–band analysis tool (GPR-BAT) [86] included in the MLRA toolbox was used as an additional method to identify the importance of spectral bands for trait estimation. For this, a GPR model was fitted using ten-fold cross validation. GPR was used with an automatic relevance determination (ARD) kernel, where correlation length scales *σ_i_* for each spectral band of the GPR covariance (kernel) functioncan be directly used as band importance measures [46,87]. Iteratively, the spectral band exhibiting the highest *σ_i_* value of the ARD kernel [47,86] was omitted from the GPR model using a sequential backward band removal (SBBR) algorithm [86] until only one spectral band remained. This resulted in an approximation of the influence of each band for the trait of interest [86]. The frequency at which a spectral band was ranked within the top five lowest Sigma values for each of the ten cross-validation folds was taken as the importance factor for said spectral band.

#### 2.2.5 Global Sensitivity Analysis

The global sensitivity analysis (GSA) toolbox was originally developed to estimate the key input variables driving the spectral output of radiative transfer models (RTM) by using sensitivity analysis of the input variables [83]. Instead of using RTM spectra, the spectra of the real-world dataset were used to perform a GSA of all sampled crop traits at once using the full FS and S2 datasets where entries for all traits were available. Contrary to the NRI regression, RFR and GPR-BAT, which are univariate analyses in which one target trait is analyzed at a time, the GSA is a multivariate analysis. Since the GSA allows estimation of the contribution of each input variable across the whole spectrum, it can be used to elicit the importance of spectral regions for all traits of interest at once [83,88]. To reduce the large computation time needed for GSA, the input spectra can be approximated using an emulator [88] that fits an ML model emulating the original spectra. Here, multiple emulators from ARTMOs MLRA toolbox were trained, and the best-performing (according to an 80/20% training/test set data split) was chosen to approximate the available spectral data. This emulator was then used to conduct the GSA, which effectively varies the target trait of interest along its variance range in a Monte-Carlo simulation, measuring the sensitivity of each spectral band to the variance change of the target trait. For each trait, 1000 iterations were simulated.

## 3 Results

### 3.1 Comparison of Spectral Analysis Methods

**Figure 1:**
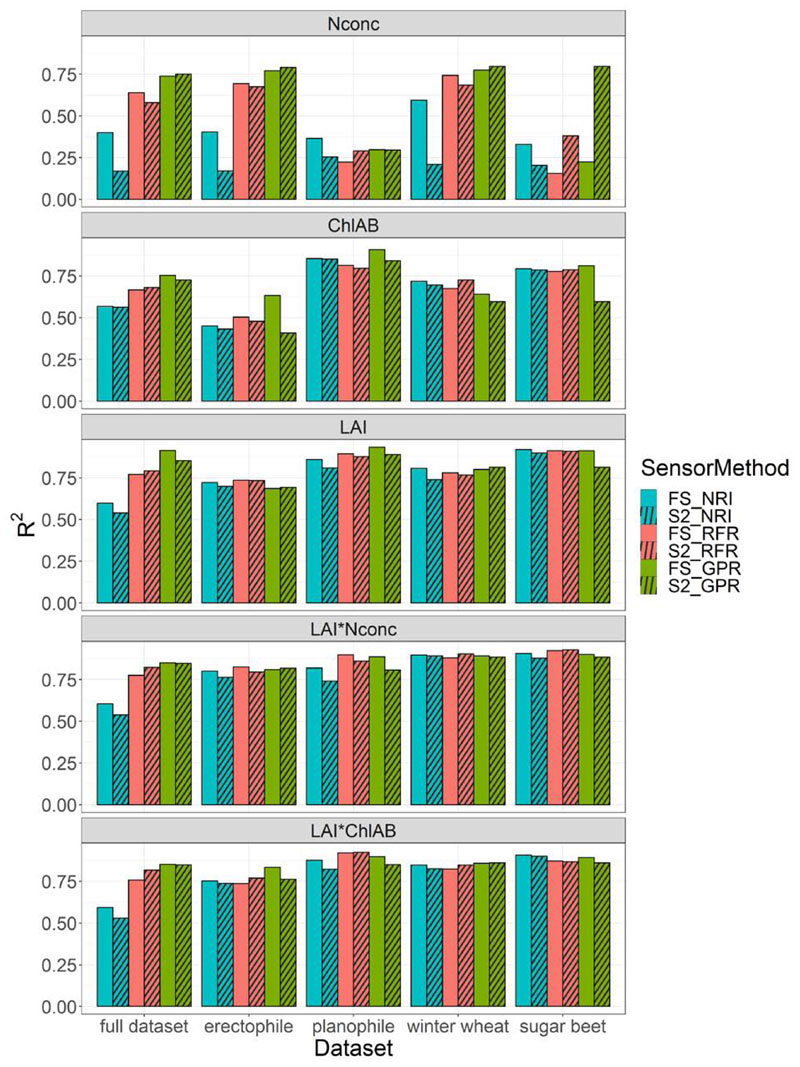
Coefficients of determination (R^2^) values for the field spectrometer (FS) dataset (empty bars) and the Sentinel-2 (S2) resampled dataset (hatched bars) for the used methods: Normalized Ratio Index (NRI, blue), Random Forest Regression (RFR, red) and Gaussian Processes Regression (GPR, green) as related to the plant traits described in Table 1.

The comparison of the NRI regression, RFR and GPR results for both the FS and the S2 dataset and all their subsets is shown in Figure 1. For N_conc_, R^2^ values for the FS datasets ranged from 0.33 to 0.59 for the NRI method (*p* < 0.001). R^2^ values for the RFR method ranged from 0.16 to 0.74 (RMSE = 0.45 to 0.52) and from 0.22 to 0.77 for the GPR method (RMSE = 0.39 to 0.47) and FS datasets. R^2^ values for the S2 datasets ranged from 0.17 to 0.25 for the NRI (*p* < 0.001); from 0.29 to 0.68 for the RFR (RMSE = 0.44 to 0.55) and from 0.30 to 0.80 for the GPR method (RMSE = 0.38 to 0.45). In the full, erectophile and winter wheat datasets, the ML-based methods RFR and GPR outperformed the NRI method for both the FS and S2 dataset. Overall, the RFR and GPR exhibited similar R^2^ values with the GPR showing slightly higher values. For the NRI method we found generally higher R^2^ values for the FS data than for the S2 resampled data. This was not observed for the RFR and GPR, where the differences in R^2^ were small. In the sugar beet dataset, both ML-based methods showed higher performance on the S2 than for the FS dataset.

For Chl_AB_, R^2^ values for the FS datasets ranged from 0.45 to 0.85 for the NRI (*p* < 0.001); from 0.5 to 0.81 for the RFR (RMSE = 0.39 to 0.66) and from 0.63 to 0.91 for the GPR (RMSE = 0.34 to 0.56). For the S2 datasets, R^2^ values ranged from 0.43 to 0.85 for the NRI (*p* < 0.001); from 0.48 to 0.80 for the RFR (RMSE = 0.35 to 0.65) and from 0.41 to 0.84 for the GPR (RMSE = 0.44 to 0.71). For each method, the differences between the FS and the S2 datasets were small except for GPR, which showed higher R^2^ values for the FS than the S2 data in the erectophile and sugar beet subsets. The NRI method performed very similarly to the ML-based methods for Chl_AB_.

For LAI, R^2^ values for the FS datasets ranged from 0.60 to 0.92 for the NRI (*p* < 0.001); from 0.74 to 0.91 for the RFR (RMSE = 0.32 to 0.81) and from 0.69 to 0.93 for the GPR (RMSE = 0.29 to 0.58). For the S2 dataset, R^2^ values ranged from 0.54 to 0.90 for the NRI (*p* < 0.001); from 0.73 to 0.91 for the RFR (RMSE = 0.30 to 0.75) and from 0.69 to 0.89 for the GPR method (RMSE = 0.35 to 0.59). Differences between the FS and the S2 data were very small for LAI, with the FS data exhibiting only slightly higher R^2^ values. Performance of the NRI was overall similar to that of the ML-based methods, except for the full dataset.

For the LAI-scaled trait LAI*N_conc_, R^2^ values for the FS data ranged from 0.61 to 0.91 for NRI (*p* < 0.001); from 0.78 to 0.92 for RFR (RMSE = 1.56 to 3.97) and from 0.81 to 0.90 for GPR (RMSE = 1.40 to 3.35) depending on the subset. For the S2 data, R^2^ values ranged from 0.54 to 0.89 for the NRI (*p* < 0.001); from 0.80 to 0.93 for the RFR (RMSE = 1.46 to 3.73) and from 0.81 to 0.89 for the GPR (RMSE = 1.47 to 3.51). Differences between the FS and S2 datasets were small.

For the LAI-scaled trait LAI*Chl_AB_, R^2^ values for the FS data ranged from 0.59 to 0.91 for the NRI (*p* < 0.001); from 0.74 to 0.92 for the RFR (RMSE = 1.86 to 5.49) and from 0.83 to 0.89 for the GPR (RMSE = 1.97 to 4.75). For the S2 data, R^2^ values ranged from 0.53 to 0.90 for the NRI (*p* < 0.001); from 0.77 to 0.92 for the RFR (RMSE = 1.67 to 5.29) and from 0.76 to 0.86 for the GPR (RMSE = 2.31 to 4.81). Differences between the FS and the S2 data were again small.

### 3.2 Spectral Band Selection

#### 3.2.1 Random Forest Variable Importance

**Figure 2:**
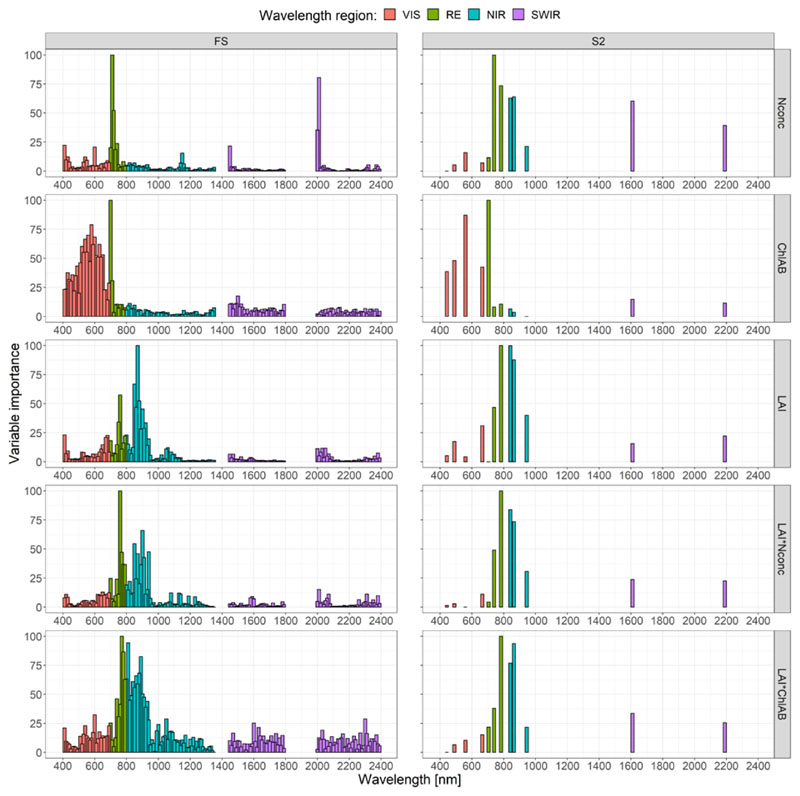
Calculated variable importance scores of the random forest regression (RFR) on the full dataset for the field spectrometer (FS, left) and the Sentinel-2 (S2, right) resampled data. The colors show the waveband regions visible (VIS: 400–690 nm), red edge (RE: 700–790 nm), near infrared (NIR: 800–1350 nm) and short-wave infrared (SWIR: 1450–2400 nm). The water absorption bands in the regions 1350–1450, 1790–1990 and >2400 nm were omitted due to their low signal to noise ratio.

The waveband ranking for N_conc_ in the full FS dataset (Figure 2, left column) showed the RE spectral region around 710 nm to be of high importance for the RFR model, along with the band at 400 nm and two bands in the SWIR region around 2000 nm. For the full S2 dataset (Figure 2, right column) the two RE bands (RE2 at 740 nm and RE3 at 783 nm, see Table 2), the two NIR bands and SWIR bands were influential for N_conc_ approximation.

The waveband ranking for Chl_AB_ on the full FS dataset showed the most influential variable to be the band at 700 nm in the RE region. The VIS region, especially the green to red domain (520 to 660 nm), contained many bands ranked with high importance. The band ranking for Chl_AB_ in the S2 dataset showed the same wavelength at 705 nm (RE1 band of S2) to be highest ranked followed by the green band at 560 nm. The other bands in the VIS range at 490, 665 and 443 nm (the S2 bands blue, red and coastal aerosol) also seemed to be important variables being less highly ranked, showing a similar pattern as observed for the FS dataset.

For LAI, in the full FS dataset, we found the NIR region at 870 nm to have the highest rank followed by other bands in the NIR, RE and one band in the VIS region at 400 nm. The S2 dataset exhibited the two bands RE3 at 783 nm and NIR1 at 842 nm as being highest ranked for LAI estimation, followed by the NIR2 band at 865 nm. Ranking for the FS dataset showed a similar pattern, where the NIR region between 850 and 870 nm and the RE region at 750 and 760 nm were shown to be the most important.

The two LAI-scaled traits showed bands in the RE and NIR regions between 760 and 940 nm to be of importance for the FS dataset. For LAI*N_conc_, the band at 760 nm was the highest ranked, followed by the band at 900 nm, showing a much lower importance. In the S2 dataset the RE3 band at 783 nm was ranked the highest, followed by the NIR1 band at 842 nm and the NIR 2 band at 865 nm being very similar as in the FS dataset.

For LAI*C_conc_ four bands in the RE (bands 770, 780 nm) and NIR region (bands 810, 890 nm) were ranked highest. For the S2 dataset, the RE3 band at 783 nm was found to be the most important, followed by the NIR1 and NIR2 bands at 842 and 865 nm, respectively. The other S2 bands possessed a much lower variable importance.

#### 3.2.2 Gaussian Processes Regression–Band Analysis Tool

**Figure 3:**
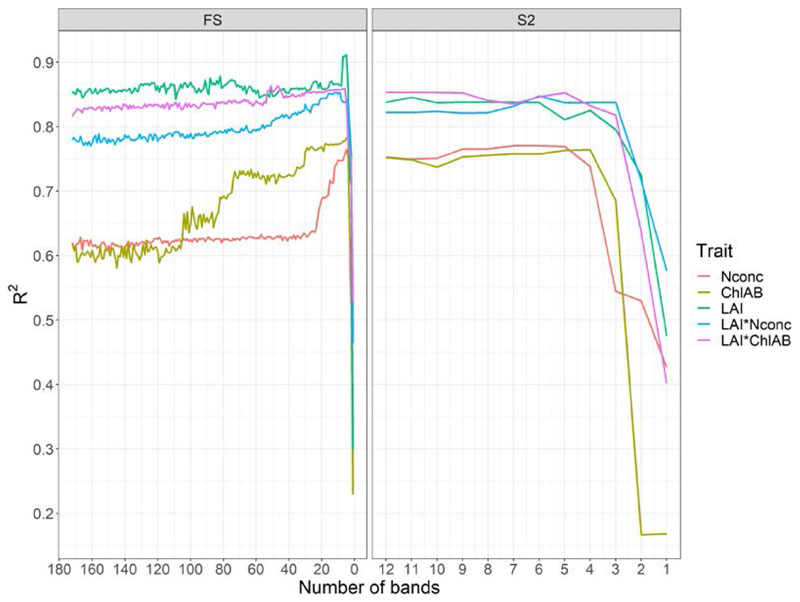
Prediction performance in (R^2^) for the traits as a function of the number of spectral bands obtained using the Gaussian processes regression–band analysis tool (GPR-BAT) tool with sequential backward band removal (SBBR) algorithm applied (for details on SBBR, see: [86]).

The GPR-BAT performed on the full FS dataset showed the LAI and LAI-scaled traits to be largely invariant to band removal until five bands were left, after which R^2^ values decreased sharply (Figure 3). Prediction performance for N_conc_ was invariant to band removal until 20 bands, after which GPR R^2^ values increased until five bands were left, after which the R^2^ values decreased sharply again. Chl_AB_ showed a similar trend, where R^2^ values increased until five bands were left and then sharply decreased. The RMSE values for the traits followed the same trend, albeit inverted. They stayed invariant to band removal (or decreased) until ten to five bands and then sharply increased (Supplementary Figure S4). GPR-BAT R^2^ values for the five most important FS bands were 0.74 for N_conc_, 0.75 for Chl_AB_, 0.91 for LAI and 0.84 for LAI* N_conc_ and LAI* Chl_AB_. For the S2 dataset, GPR-BAT R^2^ values were 0.77 for N_conc_, 0.76 for Chl_AB_, 0.81 for LAI, 0.84 for LAI*N_conc_ and 0.85 for LAI*Chl_AB_. Therefore, the top five ranked bands were used for analysis of the GPR-BAT.

**Figure 4:**
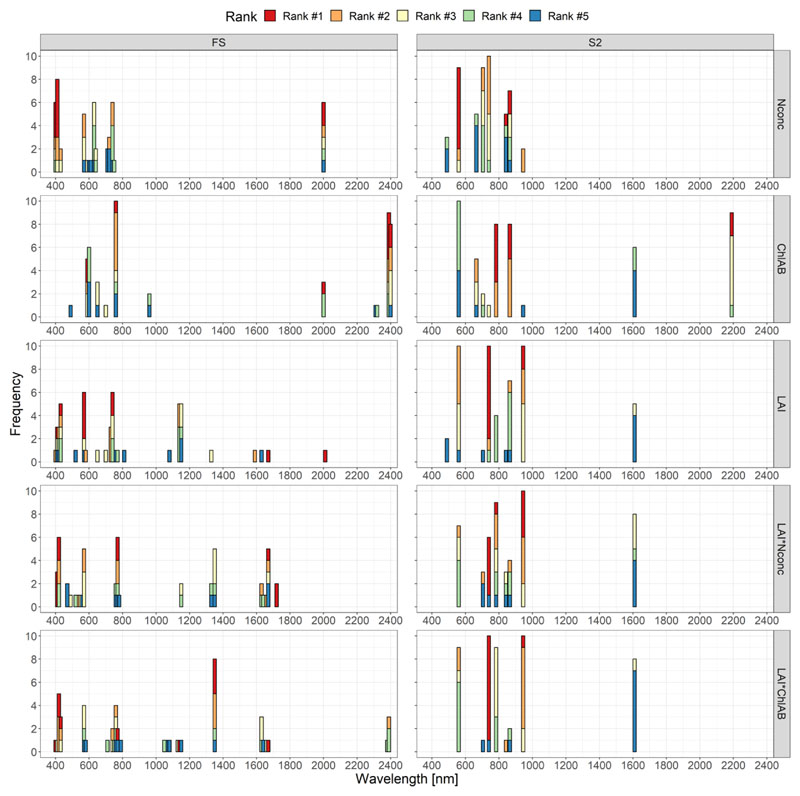
Occurrence of the top five ranked bands with lowest GPR sigma values for the ASD sensor (**left**) and the S2 resampled sensor (**right**). Data from 10-fold cross validation, e.g., 50 (10 folds × 5 ranks) is the maximum possible occurrence.

Figure 4 shows the frequency of how many times a certain band was ranked from 1st to 5th place across all the ten folds from the cross-validation performed in the GPR-BAT (see Section 2.2.4).

For the N_conc_, the VIS region around 400 nm and the SWIR region (around 2000 nm) was shown to be of high importance. The green and early red (around 600 nm) and RE (around 700 nm) regions were shown to be of minor importance for the full FS dataset. The S2 dataset showed especially the green band of S2 at 560 nm and—less often—the two NIR bands at 842 and 865 nm to be the most important bands. The S2 RE bands at 705 and 740 nm were also important, albeit ranked in the second rank.

The spectral bands with the largest importance for Chl_AB_ estimation using GPR for the FS dataset were located in the SWIR region around 2400 nm, with other important bands in the green (590 nm), RE (760 nm) and SWIR region at 2000 nm. The S2 dataset showed the most important bands to be the SWIR2 band at 2190 nm and the RE3 band at 783 nm and the NIR2 band at 865 nm. This was a slightly stronger focus on the NIR region compared to the FS dataset.

For LAI, we found a large spread of important bands over the spectrum for the FS dataset. The bands at 570 nm and at 740 nm were ranked 1st the most often. The top ranked bands were also situated in the SWIR region (once at 1670 and 2010 nm) and in the blue VIS region around 420 nm. The S2 resampled dataset showed a strong focus on the RE region, with the RE2 band at 740 nm being the first-ranked band the most often. The water vapor band at 945 nm in the NIR region also exhibited high importance. The S2 green band at 560 nm—the most important region in the FS dataset—was also highly, but not top, ranked.

The distribution of important bands for LAI*N_conc_ was like LAI for the FS dataset. The important bands were in the VIS region at 410 and 420 nm, at 770 nm in the RE region and two in the SWIR region at 1670 and 1720 nm. The S2 resampled dataset showed the RE2 band at 740 nm, the RE3 band at 783 nm and the water vapor band at 945 nm to be the most important for the GPR-BAT.

For estimation of LAI*Chl_AB_ from the FS dataset, important bands were found across the full spectrum with the most important band located at 1350 nm. Other important bands were in the VIS region near 410 nm, one band at 770 nm in the NIR and one in the SWIR region at 1670 nm. The S2 resampled dataset also showed a focus on the RE region as found for LAI and LAI*N_conc_. The importance at the end of the NIR region at 1350 nm, which was observed in the FS dataset, was not observed in the S2 dataset.

### 3.3 Global Sensitivity Analysis

Using the ARTMO toolbox [78], different MLRAs were fitted to the full dataset only as the data subsets proved to be too small, resulting in insufficient emulator performance for GSA. For the full FS dataset, a canonical correlation forest was chosen as the best performing emulator with a RMSE of 3.95 and a normalized RMSE (NRMSE) of 11.8% (reflectance values). The per-wavelength NRMSE ranged from 10% in the NIR plateau up to 17.57% at 720 nm in the RE region (Figure S5). For the S2 resampled dataset, the canonical correlation forest was also identified as the best performing emulator with a RMSE of 3.57 and NRMSE of 11.72%. The per-wavelength NRMSE values ranged from 10.26% at the NIR2 band (865 nm) to 12.66% at the RE1 band situated at 705 nm (Figure S6).

**Figure 5:**
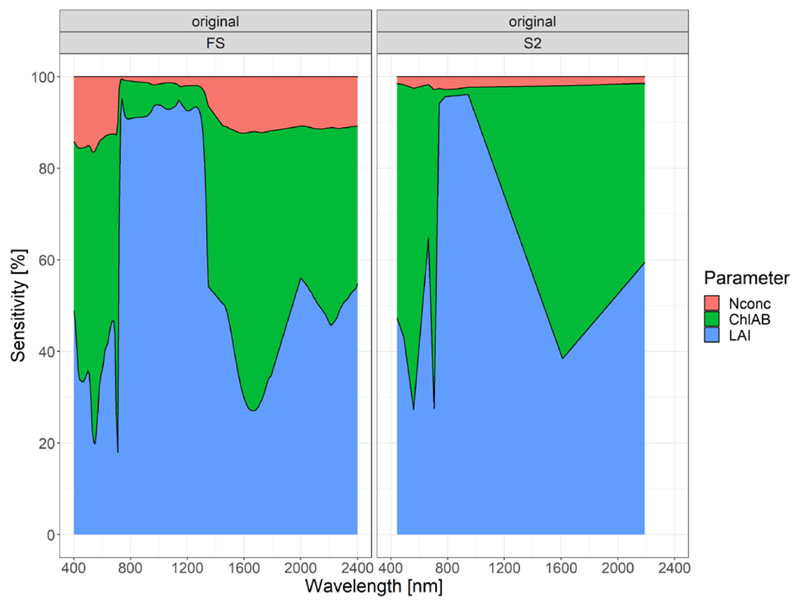
GSA results for the ASD ground spectrometer (left) and the Sentinel-2 resampled (right) sensor for the full dataset.

The sensitivity of each spectral band for the trait estimation showed LAI to be the most dominant variable, especially in the RE and the NIR region (Figure 5). The LAI showed up to 93% of the total sensitivity in these regions. This was independent of the dataset (FS and S2). LAI also exhibited strong sensitivity in the VIS region around 400 nm. Sensitivity for the LAI dropped in the SWIR region after 1400 nm but remained large with a local SWIR peak at 2000 nm. The S2 dataset showed very similar pattern for the LAI in the regions, where an S2 band was located.

The sensitivity pattern observed for Chl_AB_ was very different from the one observed for LAI, exhibiting peaks where LAI showed a low sensitivity at 710 nm (72.04%), in the green region around 550 nm (56%), and in the SWIR region around 1670 nm (61%). The lowest sensitivity was observed in the RE and NIR region, where LAI was dominant. The S2 dataset showed very similar sensitivity as the FS dataset, albeit at a much lower spectral resolution.

For N_conc_, a very low sensitivity compared to the other traits was observed, ranging from 0.5 to 16.47%. Sensitivity for N_conc_ was especially low in the RE and NIR region, where LAI was dominant. The lowest sensitivity was found at 740 nm. The wavelength region with the highest sensitivity for N_conc_ was the VIS region, with an average sensitivity of 15% and the peak sensitivity for N_conc_ of 16.47% located at 540 nm. Large parts of the SWIR region from 1400 to 2400 nm showed sensitivities ranging from 10 to 12%. For the S2 dataset, N_conc_ exhibited very low sensitivity over the whole spectrum, with values ranging from 1.4 to 2.8%.

## 4 Discussion

### 4.1 Optimal Analysis Method Depends on Target Trait

For Chl_AB_, coefficients of determination (R^2^ values) for the NRI method were in the same range or better than the Random Forest Regression (RFR) and Gaussian processes regression (GPR) approaches (Figure 1). Other studies using NRIs found significant R^2^ values for estimating crop-specific Chl_AB_ of 0.55 for winter wheat [89], 0.92 for maize, 0.81 for soybean [34] and 0.77 for sugar beet [90], comparable to the values observed for the crop-specific subsets found in this study (0.72 for winter wheat and 0.79 for sugar beet). R^2^ values found for Chl_AB_ for the full, crop unspecific dataset were, however, much lower (<0.57). This was to be expected, as the large variance between the crops is not only caused by different Chl_AB_ levels across different crops, but also by the strongly differing canopy architecture, leaf morphology and partly growth stages (Table 1). Due to the mediocre correlation between Chl_AB_ and plant N status [30], traits such as N_conc_ and LAI are more interesting from an agronomic viewpoint, since they are more directly related to the plant management decisions of the farmer on the field level.

For N_conc_, a mass-based N measure, the ML-based methods RFR and GPR performed generally better than the NRIs, a finding also confirmed by [5,91]. This was most pronounced in the full dataset, where RFR R^2^ was 0.64 (RMSE = 0.52) and GPR R^2^ 0.74 (RMSE = 0.39). This is in the same range as reported by [44] for RFR applied for pastures (R^2^ = 0.76 and RMSE of 0.38), e.g., grassland, which are mixed species stands. A slightly different approach was used by [45], who calculated SIs and did subsequent RFR for N_conc_ in winter wheat (R^2^ = 0.87 and RMSE of 0.32), e.g., a single-crop dataset. We found RFR R^2^ values through direct estimation to be 0.74 (RMSE = 0.47) for the crop-specific winter wheat dataset. Ref. [47] reported GPR R^2^ values for mass-based N (in mg g_−1_) of 0.3 ± 0.07 for a dataset of mixed tree species.

The area-based N trait LAI*N_conc_ exhibited much higher model performance than the N_conc_ in our study. This is in line with literature citing the direct estimation of N_conc_ to be mediocre [30]. However, signal separation remains an issue with this composite parameter, as described further below in the discussion of the spectral regions of interest and the GSA (see Section 4.5).

The biomass-related trait LAI was estimated better with the ML-based methods than the NRI method. Using NRIs, we explained 0.59 of the observed variation in the crop unspecific full dataset, which is less than the R^2^ of 0.71 reported by [92], who also used NRIs in a mixed-crop dataset. The R^2^ of up to 0.92 found in the crop-specific subsets was similar to single-crop values of up to 0.98 found for maize [93]. R^2^ for LAI predicted with the RFR model was 0.77 (RMSE = 0.81) for the full dataset and a maximum of 0.91 for the sugar beet dataset (RMSE = 0.32). These values are comparable to the ones found by [94] for soybean (R^2^ = 0.74 and RMSE of 0.11) and [78] for rice (R^2^ = 0.76 and RMSE of 0.67). Ref. [95] found R^2^ values for LAI in a multi-crop dataset of up to 0.91 using GPR (RMSE = 0.51). This was very similar to the results obtained in this study (full dataset R^2^ of 0.91 using GPR, RMSE = 0.55).

### 4.2 Low Specificity of Index-Based Methods for Satellite-Based Remote Sensing

The parametric, index-based methods performed very well overall, indicating that they can be readily used for proximal remote sensing tasks, e.g., with a FS. More sophisticated SIs that take either more than two bands into account [28,33,50,58], or are a composition of multiple indices [29,96], can—and regularly do—increase prediction results over NRIs. NRIs are, however, still a capable instrument in remote sensing, offering fast and efficient calculation and easy interpretation. This explains the commercial systems based on spectral indices already in operation [32,40,41]. For the S2 resampled dataset representing satellite-based remote sensing, performance of the NRIs was low, especially for N_conc_. This was mostly due to the unavailability of bands in the S2 sensor that were important in the FS dataset for N_conc_ (Figures S7 and S8). This is also reflected in the literature, where more sophisticated indices, such as MSAVI, ClRedEdge, etc., are often used for N_conc_ estimation from S2 data instead of NRIs [32,34]. These indices were also calculated in this study, but yielded very low coefficients of determination for N_conc_ on the full dataset (R^2^ of 0.02), and were sometimes not even significant. The performance on the crop-specific datasets was found to be similarly low. These non-NRI indices were shown to be not specific for traits, e.g., multiple indices showed equal prediction performance for N_conc_. The same was also observed for Chl_AB_, where the prediction performance of more specialized vegetation indices such as the MSAVI, MCARI and ClRedEdge index [33,34,89] and others showed half of the indices to be situated within <0.1 R^2^ of each other. Especially LAI and Chl_AB_ shared indices that were indicative of these two traits at the same time. This was true for both the FS and the S2 dataset. Another reason for the low specificity of the index-based methods may be the crop-unspecific dataset, as well as the few datapoints of the generative growth stages. The nonparametric ML methods RFR and GPR showed similar prediction performance between the FS and the S2 datasets (Figure 1). RFR and GPR performed especially well in the prediction for both the mass- and the area-based N trait and LAI in the S2 dataset compared to the NRIs. Coupled with the low specificity of the index-based methods, this indicates that ML-based approaches may be better suited for satellite-based remote sensing applications of plant N than index-based methods. This better performance was especially apparent in the full dataset reflecting all the heterogeneity mentioned above.

### 4.3 Model Performance on Data Subsets

In the smaller, crop-specific datasets, the relative performance of the NRI increased (Figure 1). Even though random forests (RFs) can work well on small datasets [97], they—like all ML-based methods—generally perform better with larger datasets [98]. The RFR and GPR performances on the data subsets showed large variation in R^2^ and RMSE values in the data subsets (Figures S9 and S10), which were too small to fit a reliable emulator in ARTMO and run a GSA (see Section 2.2.5). This suggested that in the case of limited data availability, the computationally far less expensive NRI method performs similar as the ML-based methods. It is important to note that this is generally only the case for crop-specific datasets, as the NRIs were outperformed by the ML methods on the unspecific full datasets containing several crops (Figure 1). This could be due to the ML-based methods using more input variables (e.g., spectral bands) than the NRI approach. RFR and GPR use all available spectral bands for regression and therefore more of the available information of the spectrum compared to the index-based methods that used two bands in this study and usually up to four bands [28,56]. It is likely that underlying crop architecture and morphology, as affected by growth stage, related background soil signal, plant health status, etc., are used by the RFR and GPR algorithms. The full dataset contains the most variance, therefore the ML models also perform the best on them. For that reason, we see the full dataset as valuable for agro-ecosystem modeling and monitoring. To fully answer these questions, especially with respect to crop-specific datasets, further data collection campaigns are needed to obtain larger crop spectral libraries (CSL) to exploit the power of ML-based algorithms in conjunction with the S2 satellites.

### 4.4 Influence of Band Number and Bandwidth on Trait Estimation

Trait estimation of the RFR and GPR methods on the S2 dataset showed little performance loss compared to the FS dataset (Figure 1), indicating that the reduced amount of S2 bands (*n* = 12) compared to the FS bands (*n* = 172) did not deteriorate model performance. This was confirmed in the GPR-BAT, where band removal kept R^2^ and RMSE values relatively stable—or even improved them—until the number of five bands was reached, after which the model performance dropped strongly (Figures 3 and S4). This indicated that GPR was able to predict the traits used in this study optimally using five spectral bands. A similar finding was reported for chlorophyll and LAI by [86], who found these traits to be optimally estimated using four to ten spectral bands. Ref. [47] found that reducing bands improved estimation of mass-based N (in mg g^−1^), but in contrast found the number of bands to be around 100.

Another reason for the good performance of the ML methods on the S2 dataset may be that these methods cope better with the broad bandwidth of certain S2 bands (Table 2), especially for certain NIR and SWIR bands. The effect of the broad bandwidth of the S2 sensor could also be observed in the band importance rankings. Especially in the case of Chl_AB_, where the S2 dataset exhibited the same important bands in the VIS range as the FS dataset, but aggregated to the four VIS bands of S2 (Figure 2). In contrast, the hyperspectral FS data showed good performance on the narrowband NRIs compared to the broad bands found on the S2 satellite. It is therefore possible to predict certain traits such as Chl_AB_ and LAI using only two narrow bands of the spectrum. This also shows that, depending on the user and the trait of interest, input variables can be reduced, saving on computation time or sensor cost.

### 4.5 Spectral Regions for Trait Estimation

In the multivariate global sensitivity analysis (GSA, Figure 5), the LAI was shown to be the most dominant variable for the spectral reflectance measurements, which confirms findings of other studies conducting GSA [65,88,99]. These studies, however, found LAI to be most important in the short-wave infrared (SWIR) region, which is a finding we could not confirm in this study, where the red edge (RE) and especially the near-infrared (NIR) regions were most important for the LAI estimation (Figures 2, 4 and 5). Since most studies employing the GSA used simulated data from RTMs, direct comparison to the real-world spectral datasets is difficult, but nevertheless very important. The GSA emulator performance measures found in this study were much lower than the emulator performed on a simulated RTM [88], but can be considered adequate for real-world data, with an NRMSE of 11.72%. The lower performance can be attributed to the full dataset containing different crop species and growth stages. Although the same FS and lab equipment were used to obtain the data of the three original datasets (Section 2.1), different people collected the data. This may have added additional variation in the dataset. Other effects such as different crop varieties, effects of biotic (pests and diseases), and abiotic stresses (nutrient and water limitation), or presence of weeds in monocrop stands may cause additional noise compared to simulated data. Such effects can never be fully avoided in real-world spectral data. Despite these limitations of the real-world dataset, the overall GSA regions for Chl_AB_, LAI and N_conc_ showed similar regions as obtained with RTM data [65,88,99], indicating both the validity of RTM and the applicability of the real-world dataset used for this study.

Univariate analyses also found the RE and NIR regions to be important for LAI estimation [52,53,55,56]. In the GSA, N_conc_ was shown to be dominated by the other traits, with only the visible (VIS), RE and SWIR region showing low sensitivity (Figure 5). This was also found in the GSA of the RTM-based studies [65,88].

For the Chl_AB_ trait, the RE region around 700 nm and the red region around 600 nm were the most important. The RFR and GPR-BAT exhibited contrasting results in band importance with the GPR-BAT exhibiting the far SWIR region around 2400 nm to be of high importance for Chl_AB_. Ref. [47] also found the far SWIR region at 2250 nm to be important for Chl_AB_ in a GPR-BAT analysis.

### 4.6 Important Bands for Plant N Estimation

In the univariate spectral region analysis on the FS dataset, the VIS region at 400 nm, the RE region at 740 nm and the SWIR region at 2000 nm were shown to be the most influential for sensing N_conc_ (Figures 2 and 4). The importance of the RE regions in plant N_conc_ estimation has been shown previously [20,24,31,100]. The VIS region for plant-N-related traits has also been shown to be important [47], but to a lesser extent. The importance of the SWIR region for mass-based plant N estimation has been shown previously [35,46]. Since large proportions of leaf N are bound in proteins [3], which exhibit high spectral absorption in the SWIR region [5,38], the importance of the SWIR bands for N_conc_ was expected. For area-based plant N, the SWIR regions were found to be important as well [46]. This finding was only partially confirmed in this study, where the spectral regions of interest for LAI*N_conc_ were primarily observed in the RE and NIR regions and only partially in the early SWIR region around 1650 nm in the GPR-BAT analysis for both the FS and the S2 dataset (Figure 4).

In the multivariate GSA on the S2 dataset, sensitivity for N_conc_ was even lower than that observed for the FS dataset (Figure 5). In the univariate band analysis, the S2 dataset for the N_conc_ trait showed the RE bands of S2 and the NIR bands to be the most important, very similar to the FS dataset. Ref. [59] also reported the S2 NIR band in conjunction with the S2 red band to be the most important for N_conc_ in winter wheat. The importance of the S2 SWIR bands for N_conc_ was mentioned by [101]. Ref. [24,60] both estimated area- (kg ha^−1^) and mass-based N (%) in winter wheat and highlighted the use of the three S2 RE bands [60] and a combination of NIR and RE bands [24]. Ref. [34] also used area-based N measurements (g m^−2^) and reported the importance of the S2 RE bands. The LAI*N_conc_ was also shown to be sensitive in the RE and NIR region for the S2 dataset, a result that is comparable to the literature [24,34,60].

### 4.7 Field Spectrometer Data for Satellite Data Simulation

The simulation of satellite data based on FS data has been evaluated in previous studies [24,34,59,101]. Of these, [24,34] performed simulation of S2 bands using an FS with wavelength up to 1000 nm. In this study, the spectral range was extended to 2500 nm, which was beneficial for mass-based N estimation due to the importance of the SWIR bands covering the protein-specific regions of N. Studies using true satellite imagery and FS data have obtained similar results in remote sensing plant N (mass- or area-based) and concluded similar spectral bands of interest for these traits [32,60,102], highlighting the robustness of the simulation approach followed in this study.

Simulation of satellite data allows modeling interactions of important crop traits and the remotely sensed signal without the need for expensive, large-scale field experiments and worries about satellite pixel site or mixed pixel effects. This is especially important for small-structured agricultural systems such as those found in Switzerland and southern Germany, where field sizes are small and inhomogeneous (e.g., with respect to trees within the field, hedges, and soil differences). Intercrops are often grown in the crop cycle between the main crops and have little coverage in national census databases. Therefore, prediction of crop traits on crop unspecific datasets is very important, particularly if ecological measures are an integral part in the agro-ecosystem policy fostering inter- or mixed cropping. Such cropping practices will cause more mixed pixels. It can be assumed that such practices will become more important and will increase in the future with Swiss and EU regulatory encouragement of ecological landscape measures [103]. A large CSL would be interesting for ‘end users’ such as governmental institutions to develop monitoring products, strategies and policies on an agro-ecosystem level. For such applications, a CSL including multiple crop species and varieties and information from different growth stages is sufficient to estimate plant-N-related traits as was shown in this study. This is an important step for possibly allowing derivation of valuable information on N flows such as the in- and output in agro-ecosystems supporting the identification of regional hotspots and support decisions and measures for mitigation.

### 4.8 Outlook on Remote Sensing of Plant N

Chl_AB_ and LAI are estimated robustly through remote sensing techniques currently in use [30,34,78,104]—a finding confirmed in this study. This comes as no surprise, as the S2 satellites were designed for vegetation monitoring [34]. Future work is needed in the domain of remote sensing plant N. Especially in small-scaled agricultural systems, the use of FS mounted on tractors [32,40] and unmanned aerial vehicles (UAVs) [20,105] has been proposed as an effective solution for data collection for plant N prediction and modeling. Based on such data, ML methods can be used more efficiently, or alternatively, advanced modeling techniques such as deep learning could be applied. These approaches already show promising performance for plant N estimation [49,60] but are heavily dependent on large quantities of data. An additional benefit of more data collection would be the creation of more specific (e.g., crops, growth stages, climate zones, etc.) datasets forming a CSL to allow better simulation of satellite sensors, advancing the modeling of N-related crop traits. Such a CSL would directly address the bottleneck of the small crop-specific data subsets in this study. These small subsets resulted in variations of ML performance, leading to unreliable prediction. Such datasets hold great potential, as the information about the plant morphology and growth stage would be included in them. A crop-specific CSL would be especially important for agronomists developing models for farmers which need the highest possible model accuracy for crop monitoring for management decisions such as N fertilization.

## 5 Conclusions

In this study, we showed the performance of parametric and nonparametric methods for two nitrogen (N) related traits: (1) the mass-based N measure, N_conc_, and (2) an area-based N measure, LAI* N_conc_ on a diverse real-world spectral library. Estimation of plant chlorophyll was shown to be robust, with few spectral bands in the red region around 600 nm and the red edge (RE) region around 700 nm, irrespective of whether a broadband satellite or narrowband hyperspectral field spectrometer (FS) was used. Plant chlorophyll was especially well estimated using normalized ratio indices (NRIs). The leaf area index (LAI) was estimated with good performance for both the ground-based FS and the satellite-based Sentinel-2 (S2) datasets containing single crops and the dataset containing a mixture of crops. LAI was better predicted using machine learning (ML) methods than NRIs. The estimation of N_conc_ was most successful using the ML algorithms random forest regression (RFR) and Gaussian processes regression (GPR) for both the hyperspectral FS and the S2 dataset. Hyperspectral devices achieved the best estimation results in the visible (VIS) region at 400 nm, especially in the RE region around 740 nm and the SWIR region around 2000 nm. The broadband S2 sensor needs the SWIR bands for good estimation performance. The ML algorithms were shown to be capable of estimating N_conc_ in the multiple crop dataset. Scaling the N_conc_ with LAI approximated the area-based plant N measure N uptake (NUP) and improved the prediction of crop N status by including the plant biomass signal. However, the separation of the biomass signal remains a challenge, and further research is needed. We therefore strongly recommend intensifying the data collection of plant-N-related traits and spectral measurements, as well as sharing available datasets and/or spectral libraries in order to monitor N in agro-ecosystems on a regional or even national scale. Such systems would facilitate more intelligent monitoring and decision support systems for agricultural policies and eventually precision farming.

## Supplementary Material

Supplementary dataset

Supplementary material

## Figures and Tables

**Table 1:** Datasets and traits used in this study.

Individual Traits	*n*	Min	Median	Max
N_conc_ [%]	322	0.68	3.46	5.32
Chl_AB_ [mg g^−1^]	194	2.28	5.34	7.11
LAI [m^2^ m^−2^]	272	0.05	2.09	8.63
LAI*N_conc_ [%]	210	0.17	7.20	41.25
LAI*Chl_AB_ [mg g^−1^]	193	0.14	11.25	56.01
**Combined Data**	***n***	**Min BBCH**	**Median BBCH**	**Max BBCH**
full dataset	180	15	30	80
erectophile	98	15	31	80
planophile	55	15	22	67
winter wheat	64	15	30	32
sugar beet	45	15	21	38

**Table 2:** The specifications of the Multispectral Instrument (MSI) on board the Sentinel-2 (S2) satellites (reproduced from the European Space Agency ESA). Band B10 was not used in the S2 resampled dataset as it lies within a region of atmospheric water absorption.

Band	Band Name	Center Wavelength [nm]	Bandwidth [nm]	Ground Resolution [m]
B01	Coastal aerosol	443	21.00	60
B02	Blue	490	66.00	10
B03	Green	560	36.00	10
B04	Red	665	31.00	10
B05	RE1	705	15.50	20
B06	RE2	740	15.00	20
B07	RE3	783	20.00	20
B08	NIR1	842	106.00	10
B8a	NIR2	865	21.50	20
B09	Water vapour	945	20.50	60
B10	SWIR—cirrus	1375	30.50	60
B11	SWIR1	1610	92.50	20
B12	SWIR2	2190	180.00	20

## Data Availability

The data presented in this study are openly available in: https://doi.org/10.3929/ethz-b-000488405. Please also see the ‘supplementary materials – dataset’ for more information on the dataset.
