# Supplementary material for: Crop Nitrogen Retrieval Methods for Simulated Sentinel-2 Data Using In-Field Spectrometer Data
Source: Remote Sens (Basel). Author manuscript; Available in PMC 2022 Sep 7. (PMC7613346; doi:10.3390/rs13122404)
Supplement: Supplementary dataset [file EMS152668-supplement-Supplementary_dataset.pdf]

remote sensing

# Supplementary Materials – Dataset

The following document gives additional information on the dataset used in the paper "Crop nitrogen retrieval methods for simulated Sentinel-2 data using in-field spectrometer data". Details on the sensors and how the data was collected can be found in the materials & methods part of the article. The locations of the datapoints are displayed in Figure 1. Figure 2 displays the average temperature and rainfall for the measurement region. Table 1 lists the number of different crops in the dataset. Figure 3 gives the exact BBCH growth stages of the measurements. Please note that since the dataset originates from three different projects/field campaigns, not all entries have measurements for all traits. The dataset can be downloaded from: https://doi.org/10.3929/ethz-b-000488405.

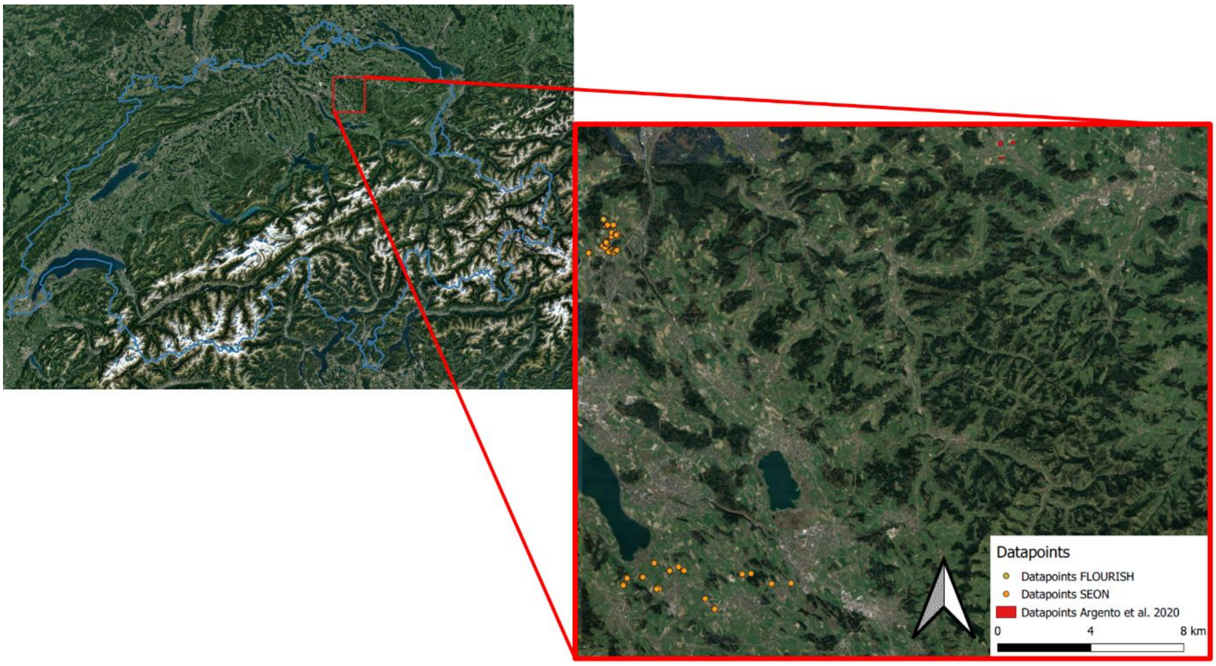


**Figure S1.** Location of the measurements where data was sampled for this paper.

| | BBCH | | | | | | | | | | | | | | | | | | | |
|---|---|---|---|---|---|---|---|---|---|---|---|---|---|---|---|---|---|---|---|---|
| Crop | 15 | 16 | 17 | 18 | 20 | 21 | 22 | 23 | 24 | 27 | 28 | 29 | 30 | 31 | 32 | 35 | 36 | 37 | 38 | 39 |
| buckwheat | 0 | 0 | 0 | 0 | 0 | 0 | 0 | 0 | 0 | 0 | 0 | 0 | 0 | 0 | 0 | 0 | 0 | 0 | 0 | 0 |
| corn | 0 | 4 | 11 | 8 | 0 | 0 | 0 | 0 | 0 | 0 | 0 | 0 | 0 | 0 | 0 | 1 | 2 | 4 | 1 | 0 |
| perm_grassland | 0 | 0 | 0 | 0 | 0 | 0 | 0 | 0 | 0 | 0 | 0 | 0 | 0 | 0 | 0 | 0 | 0 | 0 | 0 | 0 |
| potatoes | 0 | 0 | 0 | 0 | 0 | 0 | 0 | 0 | 0 | 0 | 0 | 0 | 0 | 0 | 0 | 0 | 0 | 0 | 0 | 4 |
| sugar_beet | 2 | 11 | 4 | 1 | 8 | 9 | 7 | 7 | 7 | 1 | 1 | 0 | 0 | 0 | 1 | 2 | 0 | 0 | 1 | 3 |
| summer_barley | 0 | 0 | 0 | 0 | 0 | 0 | 0 | 0 | 0 | 0 | 0 | 0 | 0 | 0 | 0 | 0 | 0 | 0 | 0 | 0 |
| summer_wheat | 0 | 0 | 0 | 0 | 0 | 0 | 0 | 0 | 0 | 0 | 0 | 0 | 0 | 0 | 1 | 0 | 0 | 0 | 0 | 0 |
| sunflower | 0 | 0 | 0 | 0 | 0 | 0 | 0 | 0 | 0 | 0 | 0 | 0 | 0 | 0 | 0 | 0 | 0 | 0 | 0 | 0 |
| temp_grassland | 0 | 0 | 0 | 0 | 0 | 0 | 0 | 0 | 0 | 0 | 0 | 0 | 0 | 0 | 0 | 0 | 0 | 0 | 0 | 0 |
| winter_barley | 1 | 0 | 0 | 1 | 0 | 0 | 0 | 0 | 0 | 0 | 0 | 0 | 0 | 0 | 0 | 0 | 0 | 0 | 0 | 0 |
| winter_wheat | 4 | 0 | 0 | 0 | 0 | 0 | 0 | 0 | 0 | 0 | 0 | 6 | 31 | 67 | 11 | 0 | 0 | 0 | 0 | 52 |

| | BBCH | | | | | | | | | | | | | | | | | | | | | | |
|---|---|---|---|---|---|---|---|---|---|---|---|---|---|---|---|---|---|---|---|---|---|---|---|
| Crop | 40 | 41 | 45 | 48 | 50 | 51 | 52 | 53 | 54 | 55 | 58 | 59 | 63 | 64 | 65 | 66 | 67 | 68 | 75 | 76 | 79 | 80 | 85 |
| buckwheat | 0 | 0 | 0 | 0 | 0 | 0 | 0 | 0 | 0 | 0 | 0 | 0 | 0 | 0 | 8 | 0 | 0 | 0 | 0 | 0 | 0 | 0 | 0 |
| corn | 0 | 0 | 0 | 0 | 0 | 0 | 4 | 4 | 0 | 0 | 0 | 4 | 0 | 0 | 0 | 0 | 0 | 0 | 3 | 1 | 1 | 3 | 0 |
| perm_grassland | 0 | 0 | 0 | 0 | 0 | 0 | 0 | 0 | 0 | 0 | 0 | 0 | 0 | 0 | 0 | 0 | 0 | 0 | 0 | 0 | 0 | 0 | 0 |
| potatoes | 0 | 2 | 1 | 0 | 0 | 0 | 0 | 0 | 0 | 0 | 0 | 0 | 1 | 1 | 2 | 1 | 1 | 0 | 0 | 0 | 0 | 0 | 0 |
| sugar_beet | 0 | 0 | 0 | 0 | 0 | 0 | 0 | 0 | 0 | 0 | 0 | 0 | 0 | 0 | 0 | 0 | 0 | 0 | 0 | 0 | 0 | 0 | 0 |
| summer_barley | 0 | 0 | 6 | 3 | 2 | 4 | 1 | 0 | 0 | 0 | 0 | 0 | 0 | 0 | 0 | 8 | 0 | 4 | 0 | 0 | 4 | 0 | 4 |
| summer_wheat | 0 | 0 | 0 | 0 | 0 | 0 | 0 | 0 | 0 | 0 | 0 | 0 | 0 | 0 | 0 | 0 | 0 | 0 | 0 | 0 | 0 | 0 | 0 |
| sunflower | 4 | 0 | 0 | 0 | 0 | 0 | 4 | 0 | 0 | 0 | 0 | 0 | 4 | 0 | 0 | 0 | 0 | 0 | 0 | 0 | 0 | 0 | 0 |
| temp_grassland | 0 | 0 | 0 | 0 | 0 | 0 | 0 | 0 | 0 | 0 | 0 | 0 | 0 | 0 | 0 | 0 | 0 | 0 | 0 | 0 | 0 | 0 | 0 |
| winter_barley | 0 | 0 | 0 | 0 | 0 | 0 | 1 | 0 | 1 | 1 | 2 | 3 | 0 | 0 | 0 | 0 | 0 | 0 | 0 | 0 | 0 | 0 | 0 |
| winter_wheat | 0 | 0 | 0 | 0 | 0 | 0 | 0 | 0 | 0 | 0 | 0 | 0 | 0 | 0 | 0 | 0 | 0 | 0 | 0 | 0 | 0 | 0 | 0 |

**Figure S2.** Detailed BBCH stages of the dataset.

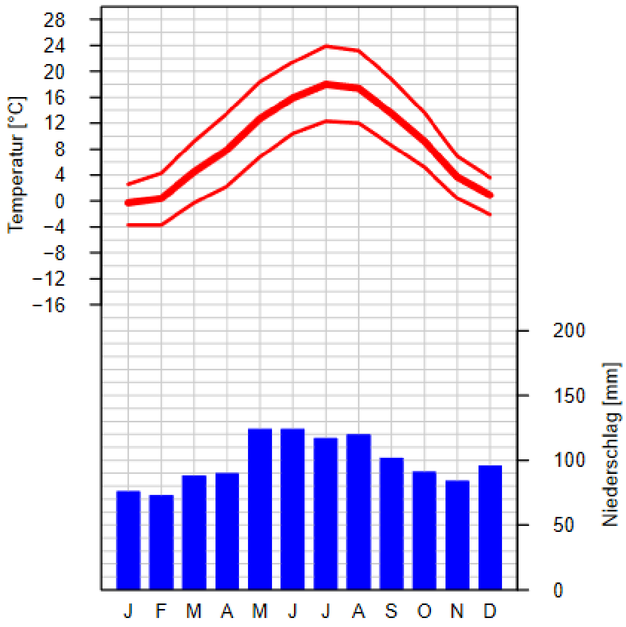


**Figure S3.** Climate diagram showing average temperature and precipitation for the region where the data was collected.

**Table S1.** The dataset contains the following 11 Crops.

| Crop | N |
|---|---|
| winter_wheat | 171 |
| sugar_beet | 65 |
| corn | 51 |
| summer_barley | 36 |
| buckwheat | 20 |
| potatoes | 13 |
| sunflower | 12 |
| temp_grassland | 10 |
| winter_barley | 10 |
| perm_grassland | 3 |
| summer_wheat | 1 |
