# Supplementary material for: Crop Nitrogen Retrieval Methods for Simulated Sentinel-2 Data Using In-Field Spectrometer Data
Source: Remote Sens (Basel). Author manuscript; Available in PMC 2022 Sep 7. (PMC7613346; doi:10.3390/rs13122404)
Supplement: Supplementary material [file EMS152668-supplement-Supplementary_material.pdf]

remote sensing

Supplementary Materials

1.1. GPR-BAT

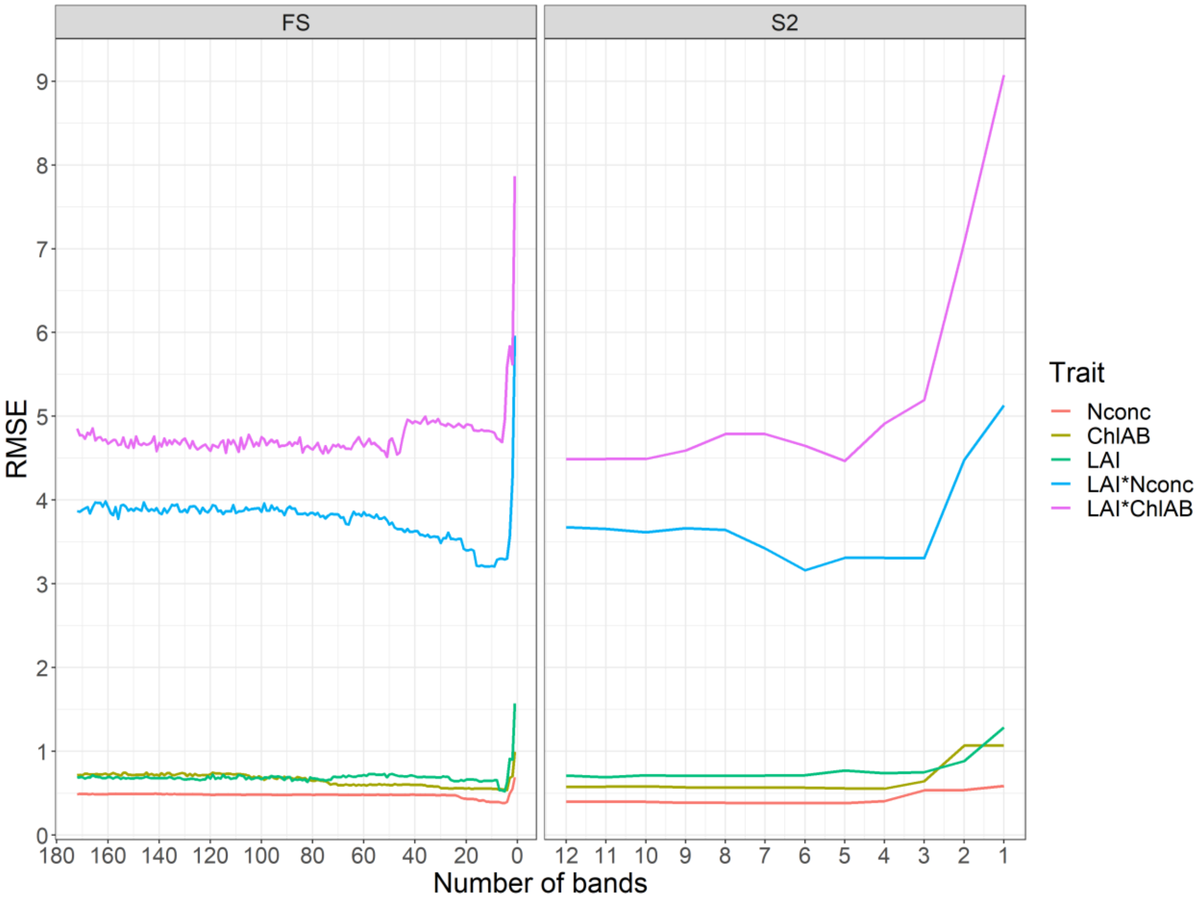

Figure S4. RMSE values of the traits as a function of the number of spectral bands obtained using the Gaussian processes regression - band analysis tool (GPR-BAT) with the sequential backward band removal (SBBR) algorithm applied.

1.2. GSA emulator performance for ASD and S2

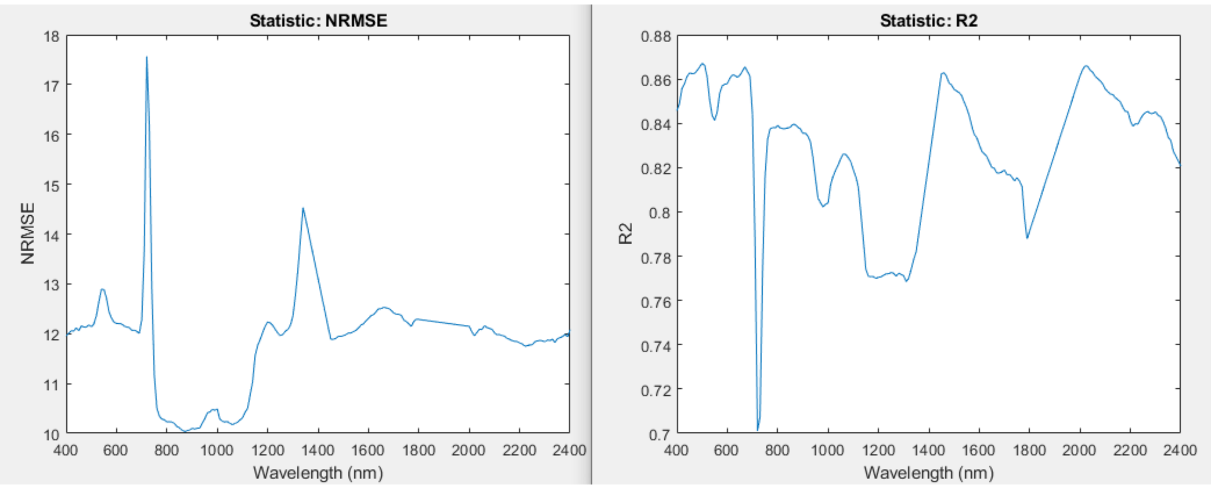

Figure S5. Performance of the emulator (Canonical Correlation Forest) on the full FS dataset with 20 PCA and 80/20% train/test set split.

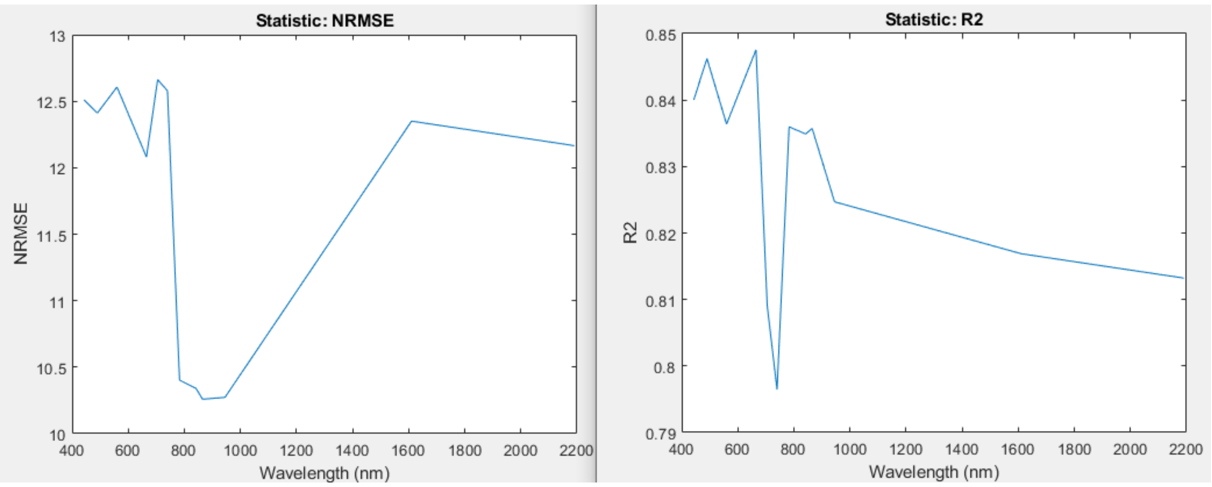

Figure S6. Performance of the emulator (Canonical Correlation Forest) on the S2 resampled full dataset with 20 PCA and 80/20% train/test set split.

1.3. NRI heatmaps

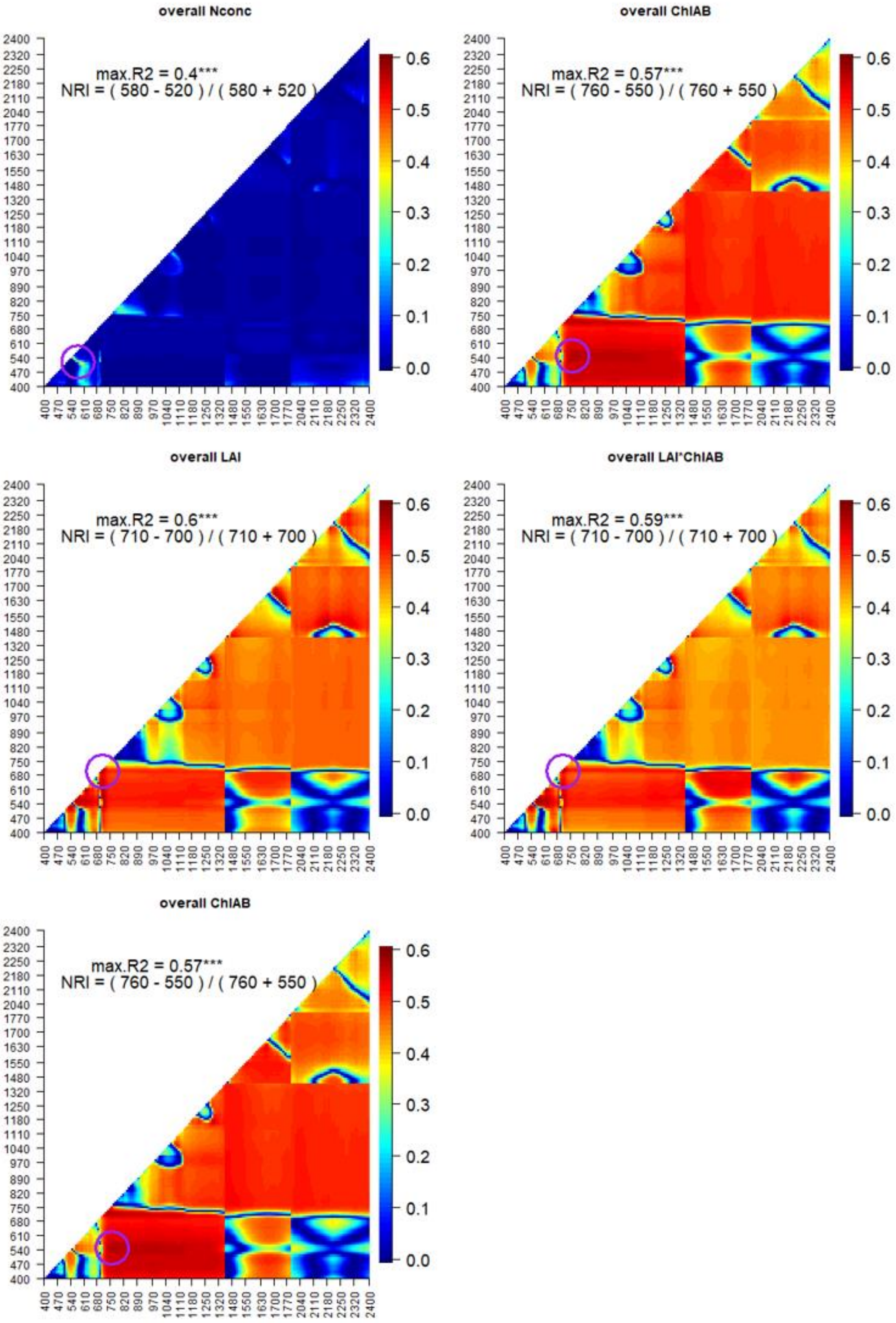

Figure S7. R2 values between the trait of interest and the normalized ratio indices (NRI's) using all possible band combinations of the full field spectrometer dataset.

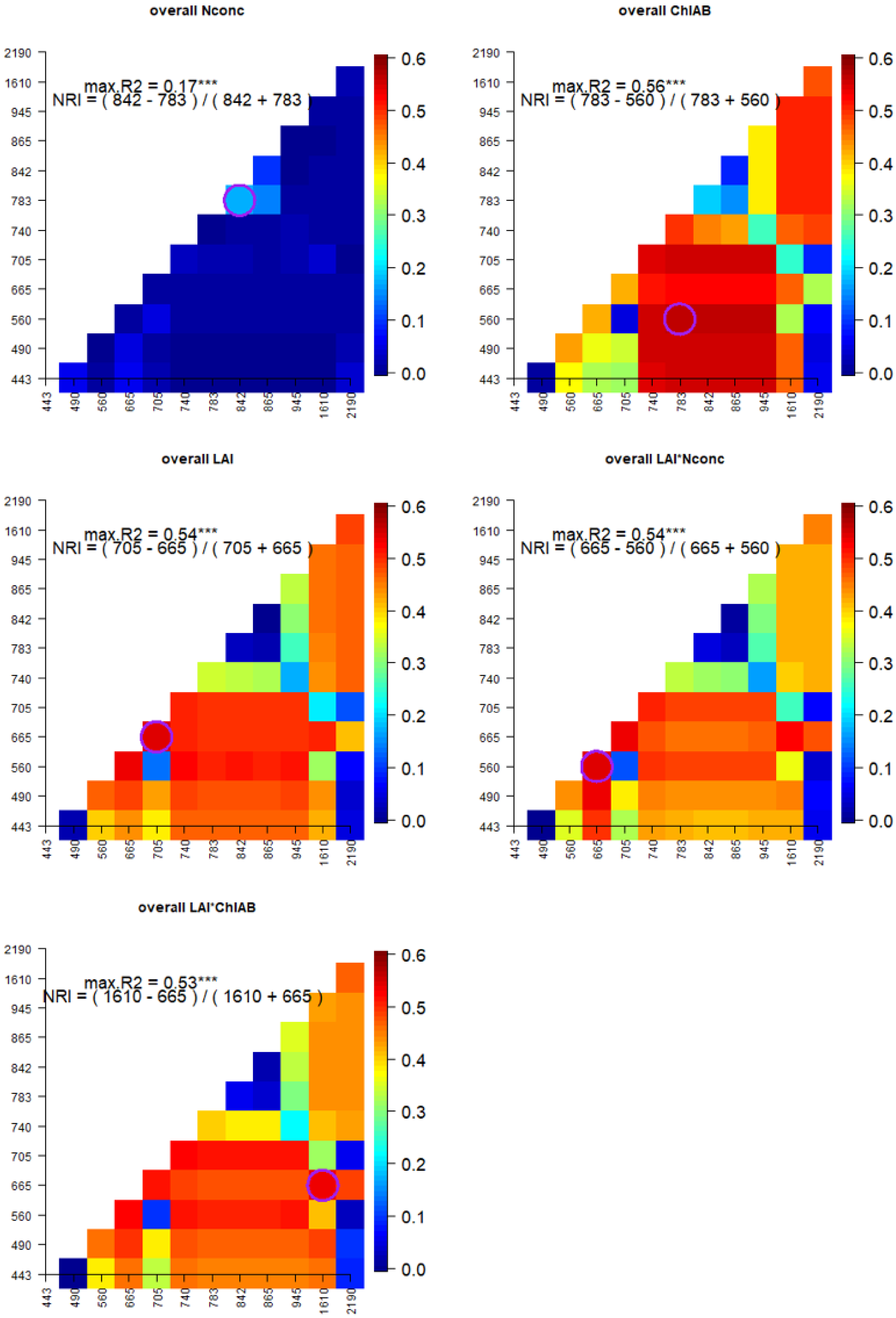

Figure S8. R^2 values between the trait of interest and the normalized ratio indices (NRI's) using all possible band combinations of the full Sentinel-2 resampled dataset.

1.4. Effect of train/test set split on RFR

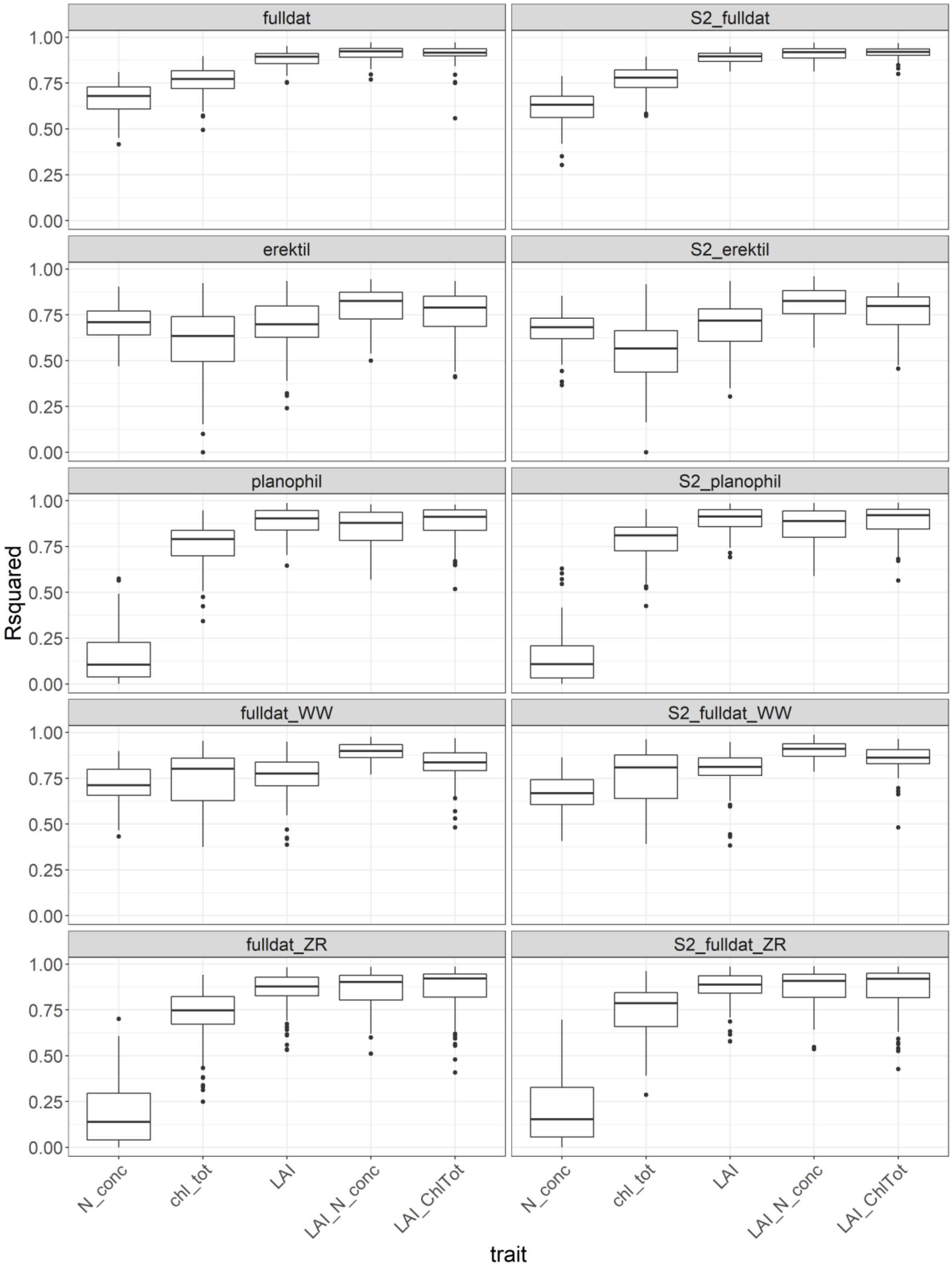

Figure S9. R2 values for prediction on the test set for 100 iterations of random train/test set splits. Train/test set splits were performed by stratification by crop and date combinations.

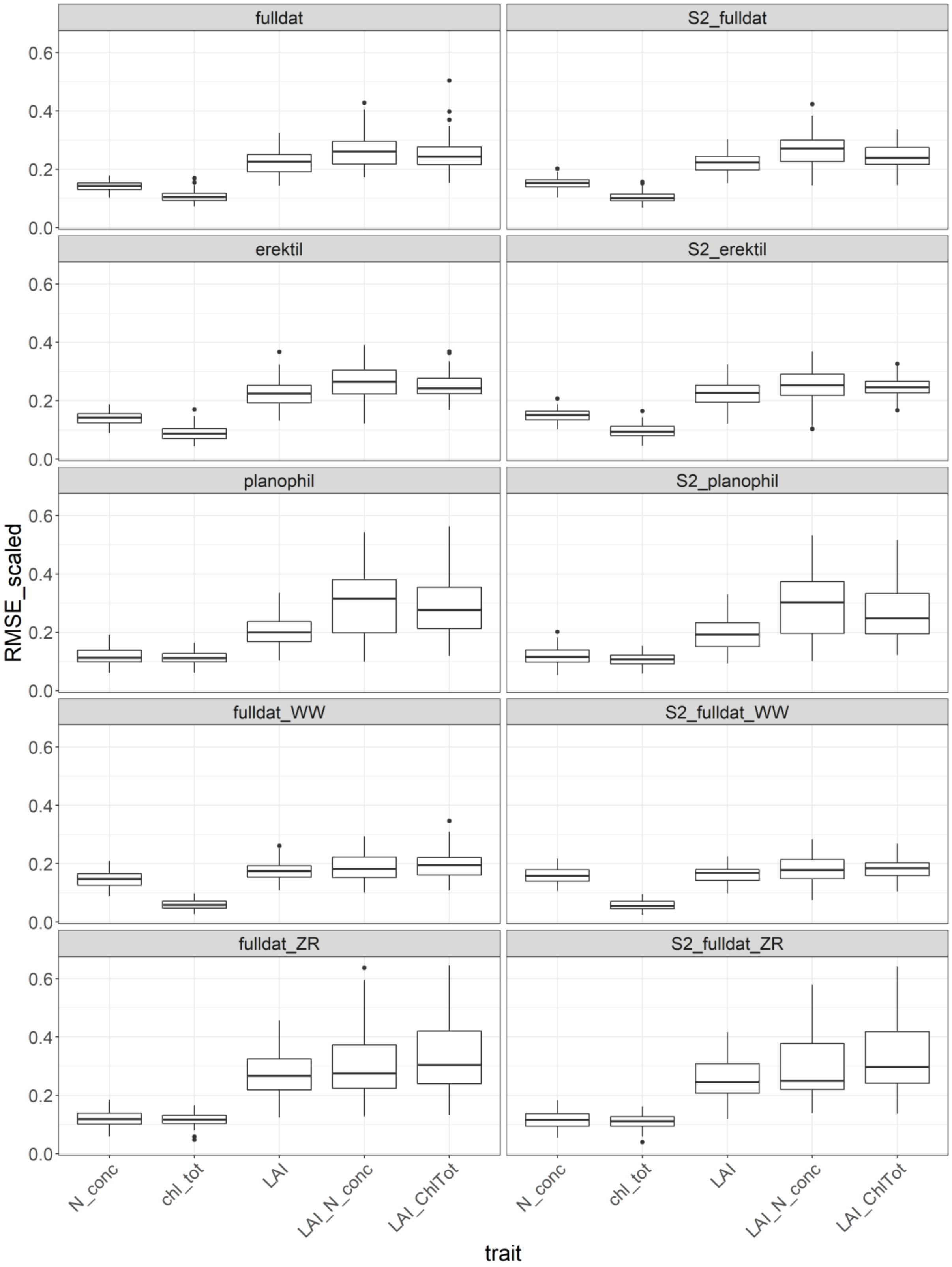

Figure S10. Median centred RMSE values for each trait & dataset combination for the same 100 iterations of random train/test set splits as found in Figure S9.
